# Comparative Transcriptome Analysis Reveals the Genetic Basis of Skin Color Variation in Common Carp

**DOI:** 10.1371/journal.pone.0108200

**Published:** 2014-09-25

**Authors:** Yanliang Jiang, Songhao Zhang, Jian Xu, Jianxin Feng, Shahid Mahboob, Khalid A. Al-Ghanim, Xiaowen Sun, Peng Xu

**Affiliations:** 1 CAFS Key Laboratory of Aquatic Genomics and Beijing Key Laboratory of Fishery Biotechnology, Centre for Applied Aquatic Genomics, Chinese Academy of Fishery Sciences, Beijing, China; 2 Henan Academy of Fishery Sciences, Zhengzhou, Henan, China; 3 Department of Zoology, College of Science, King Saud University, Riyadh, Saudi Arabia; University of Tennessee, United States of America

## Abstract

**Background:**

The common carp is an important aquaculture species that is widely distributed across the world. During the long history of carp domestication, numerous carp strains with diverse skin colors have been established. Skin color is used as a visual criterion to determine the market value of carp. However, the genetic basis of common carp skin color has not been extensively studied.

**Methodology/Principal Findings:**

In this study, we performed Illumina sequencing on two common carp strains: the reddish Xingguo red carp and the brownish-black Yellow River carp. A total of 435,348,868 reads were generated, resulting in 198,781 assembled contigs that were used as reference sequences. Comparisons of skin transcriptome files revealed 2,012 unigenes with significantly different expression in the two common carp strains, including 874 genes that were up-regulated in Xingguo red carp and 1,138 genes that were up-regulated in Yellow River carp. The expression patterns of 20 randomly selected differentially expressed genes were validated using quantitative RT-PCR. Gene pathway analysis of the differentially expressed genes indicated that melanin biosynthesis, along with the Wnt and MAPK signaling pathways, is highly likely to affect the skin pigmentation process. Several key genes involved in the skin pigmentation process, including *TYRP1*, *SILV*, *ASIP* and *xCT*, showed significant differences in their expression patterns between the two strains.

**Conclusions:**

In this study, we conducted a comparative transcriptome analysis of Xingguo red carp and Yellow River carp skins, and we detected key genes involved in the common carp skin pigmentation process. We propose that common carp skin pigmentation depends upon at least three pathways. Understanding fish skin color genetics will facilitate future molecular selection of the fish skin colors with high market values.

## Introduction

Coloration is one of the most diverse phenotypic traits in vertebrates, and it exerts multiple adaptive functions, such as species identification, camouflage, warning or threatening of predators, photoprotection, thermoregulation and photoreception [1]. Skin coloration is the result of diverse pigments synthesized by pigment cells or chromatophores, and it is affected by multiple factors, including environmental, nutritional, physiological, or genetic conditions. Among these factors, the most fundamental and important is the genetic basis of skin pigmentation: which genes are likely to be involved and how. Cellular, genetic and genomic approaches have been widely adopted to answer this question.

In humans and mammals, the pigment melanin is the primary determinant of skin color. The availability of the complete human genome sequence and of adequate genomic resources, along with genome-wide association studies (GWAS), has provided insight into the genetic basis of the pigmentation process. In contrast to mammals, which possess only one type of pigment cell (the melanocyte), and amphibians and reptiles, which possess xanthophores, erythrophores and reflecting iridophores, teleost fish possess up to six different pigment cells, including melanophores, xanthophores, erythrophores, iridophores, leucophores and cyanophores [2]. A diversity of pigment cells, associated with a series of cellular, physiological, genetic and environmental factors, makes fish skin pigmentation a complicated biological process. Extensive studies have been conducted on model fish species such as zebrafish and medaka in an effort to unravel the genetic basis of fish skin pigmentation, and dozens of genes have been reported to be involved in the pigmentation process, such as *matp*, *oca4*, *sox10*, *kit*, *ednrb*, *slc24a5* and many others, through collecting and identifying the pigmentation mutations [3]–[5]. However, few genetic skin color studies have been conducted on non-model fish species.

The common carp (*Cyprinus carpio*), a freshwater fish that is especially widespread in Europe and Asia, was domesticated more than 2,000 years ago. Over its long history of domestication, the common carp has been introduced into various environments worldwide, resulting in hundreds of strains or varieties that display rich biodiversity, genetic polymorphisms and diverse skin colors. Skin color is an important economic trait for common carp, as it acts as an important criterion for visually determining quality and market value. Several studies have provided initial insights into the genetic basis of skin coloration in common carp. For instance, David et al. reported that the *MC1R* gene was associated with the development of black pigmentation in the ornamental Koi common carp [6]. A very recent study reported that 80% of identified zebrafish pigmentation genes were also present in the Oujiang color common carp strain [7]. In addition to the direct effects of genes, other regulators, such as microRNAs, play crucial roles in common carp skin coloration by regulating the expression of downstream pigmentation genes [8]. However, some details of the genetic mechanisms underlying common carp skin pigmentation are not well understood, such as the interactions among pigmentation genes and the genetic regulation underlying synthesis of different pigments.

The Xingguo red carp (*Cyprinus carpio* var. *xingguonensis*, XGC) and the Yellow River carp (*Cyprinus carpio haematopterus* Temminck et Schlegel, YRC) are two traditional domestic strains of common carp in China. XGC, a regional strain from Xingguo County in the Jiangxi Province in Southern China, is known for its red color and has approximately 1,300 years of cultural history [9]. YRC, a brownish-black common carp, was originally cultivated along the Yellow River basin thousands of years ago and is now a prominent strain in Northern China. It possesses advantageous traits such as strong cold tolerance, high efficiency of food conversion and good meat quality. The distinct skin colors of these two common carp strains make them suitable models for exploring the genetic basis of skin pigmentation. To better understand skin color genetics, we utilized the powerful approach of comparative transcriptome analysis using next-generation sequencing and examined transcript profiles from the skins of the XGC and YRC common carp strains. We obtained candidate genes that may be involved in the skin pigmentation process and identified gene pathways that may regulate the synthesis of different pigments. Understanding the molecular mechanisms of skin pigmentation in common carp will advance our knowledge of skin color genetics in fish and accelerate the molecular selection of fish species with consumer-favored skin colors.

## Results and Discussion

### Sequencing of short expressed reads from XGC and YRC

Diverse skin colors make fish good genetic models for understanding the skin pigmentation process. Various fish colorations are determined by the density and position of different pigment cells, which is believed to be primarily under genetic control. To better understand fish skin color genetics, we conducted a comparative transcriptome analysis between two common carp strains, XGC and YRC, using next-generation sequencing. First, we generated reference sequences for the subsequent analysis of skin genes that are differentially expressed between the two carp strains. Six tissues, including brain, blood, gill, head kidney, muscle and skin were collected and deep-sequenced using Illumina HiSeq 2000. As shown in Table 1, a total of 435 million paired-end reads were generated, of which 211 million were from YRC and 224 million were from XGC. The number of reads generated from each tissue ranged from 27.5 million to 36.9 million, with 2 outliers of 50.5 million and 46.3 million from XGC muscle and blood, respectively. After the removal of ambiguous nucleotides, low-quality sequences (Q<20) and short reads (length<15 bp), a total of 422 million clean reads (97%) were selected for further analysis.

**Table 1:** Summary of the raw sequencing data from YRC and XGC.

Fish strain	Tissue	Reads	Clean reads	Mapped reads	Mapping ratio (%)
YRC	Blood	35,114,692	34,267,222	28,563,473	83
	Brain	36,058,166	35,120,875	27,380,041	78
	Gill	35,941,220	35,016,622	27,700,985	79
	Head kidney	30,353,832	29,523,567	24,663,257	84
	Muscle	36,496,782	35,529,894	31,392,686	88
	Skin	36,899,438	35,809,741	30,326,936	85
XGC	Blood	46,263,852	44,653,725	36,803,528	82
	Brain	27,463,132	26,820,218	20,751,409	77
	Gill	35,395,640	34,145,259	28,044,263	82
	Head kidney	32,929,374	31,705,811	26,548,216	84
	Muscle	50,520,326	48,807,426	43,829,803	90
	Skin	31,912,414	30,944,239	27,123,074	88

### Reference sequence assembly and annotation

All of the clean reads were pooled and *de* *novo* assembled to generate reference sequences using the Trinity assembler [10]. After the removal of sequence redundancy using CD-hit software [11], a total of 198,781 contigs, with a minimum length of 200 bp, a maximum length of 26,217 bp and an N50 of 1,970 bp, were generated as the reference sequences for subsequent analysis (Table 2). There were 43,310 contigs longer than 1,000 bp. To assess the quality of the sequencing and *de* *novo* assembly, all of the clean reads were mapped back to the assembled sequences. As shown in Table 1, the mapping ratio ranged from 77% to 90% with an average of 83%, indicating a high-quality sequence.

**Table 2:** Statistics of reference sequence assembly.

**Assembly**	No. of contigs (>200 bp)	198,781
	No. of larger contigs (>1000 bp)	43,310
	Maximum length (bp)	26,217
	N50 (bp)	1,970
	Average length (bp)	865
**Annotation**	No. of contigs with Blast hit to *nr*	62,343
	No. of contigs with Blast hit to UniProt	57,083
	No. of contigs with Blast hit to zebrafish protein	69,634
	No. of unique genes predicted	20,028
	No. of contigs with GO terms	58,841
	No. of unique genes with GO terms	16,849

Gene prediction was performed on the assembled contigs using BlastX searches against three protein databases, including the NCBI non-redundant (nr) database, the UniProt database and the Ensembl zebrafish protein database, with an E-value cutoff of 1e^−10^. There were 62,343, 57,083, and 69,634 assembled contigs with significant hits against nr, UniProt and zebrafish, respectively (Table 2). Cumulatively, 73,928 assembled contigs had at least one significant hit against at least one of the three databases, allowing for the prediction of 20,028 unique genes.

**Figure 1:**
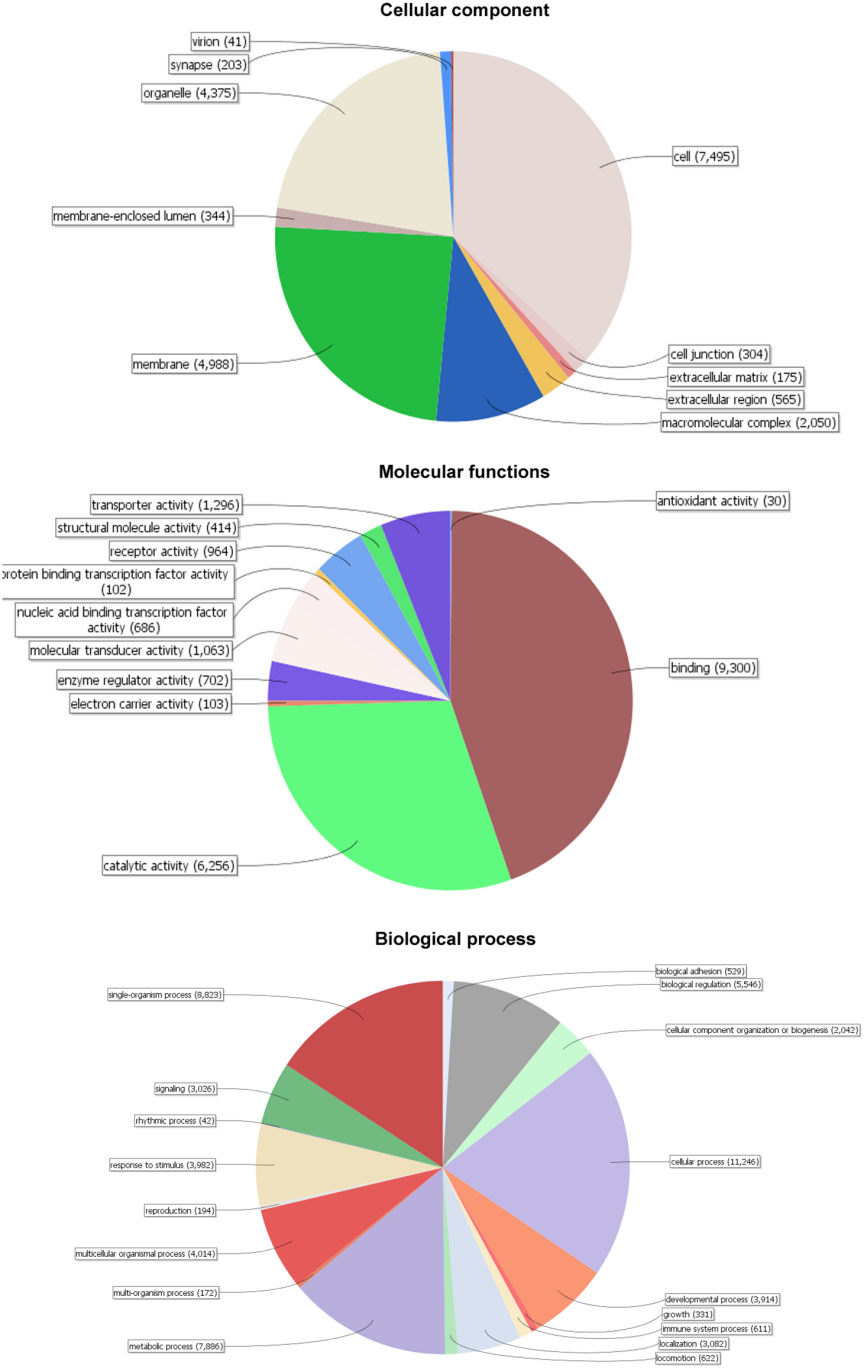
Distribution of the most common GO term categories.

Gene ontology (GO) annotation was then performed with these 73,928 unique gene-containing contigs using Blast2GO [12]. Of these, 58,841 contigs, corresponding to 16,849 unique genes, were assigned to at least one GO term (Table 2). As shown in Figure 1, a total of 38 GO terms were assigned, including 10 (26.3%) cellular component terms, 11 (28.9%) molecular function terms and 17 (60.7%) biological process terms. From the GO category of molecular function, binding was the most predominant term, accounting for 63.4% of the sequences annotated in that term, and it was followed by catalytic activity. In the biological processes category, cellular process was the most predominant term (20.2% of the sequences), and it was followed by single-organism process (16.6%) and metabolic process (14.9%).

### Identification of differentially expressed genes in XGC skin compared with YRC skin

**Figure 2:**
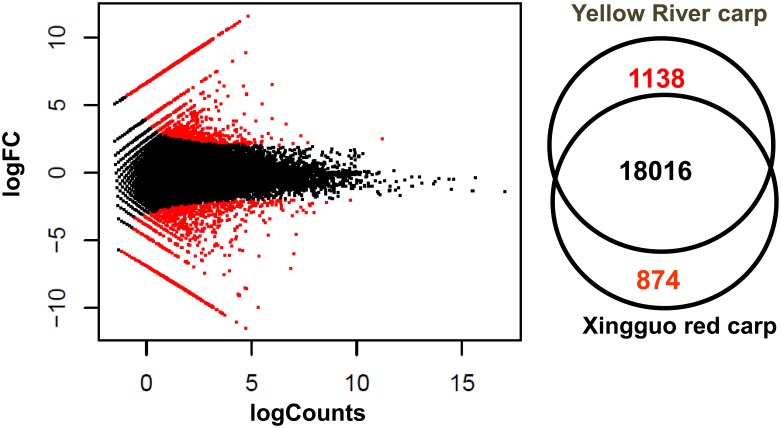
Gene expression in the skin of two common carp strains. The left panel shows an M-A plot, where the y-axis represents the logarithm of fold change and the x-axis represents the logarithm of transcript counts. The right panel shows a Venn diagram with the number of differentially expressed genes in the two carp strains.

**Figure 3:**
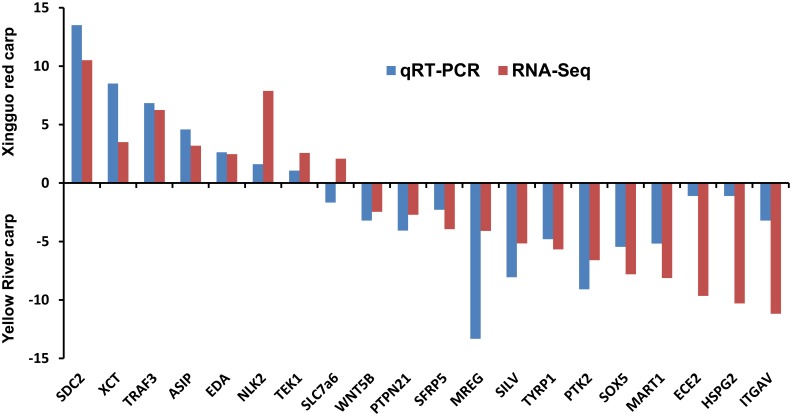
Comparison of gene expression patterns obtained using comparative transcriptome analysis and qRT-PCR. Fold changes are expressed as the ratio of gene expression between XGC and YRC after normalization to β-actin.

Based on the criteria that |fold-change|≧2 and p-value≦0.05, a total of 4,367 of the 198,781 assembled contigs showed significantly different expression in XGC skin compared with YRC skin. Differentially expressed contigs represented 2,012 unique genes, of which 874 genes were up-regulated in XGC and 1,138 genes were down-regulated (Figure 2, Table S1). To validate the differentially expressed genes identified by comparative transcriptome analysis, we randomly selected 20 representative genes for qRT-PCR confirmation of differential expression. The gene expression patterns in the result of qRT-PCR were compared with the data obtained from the comparative transcriptome analysis. As shown in Figure 3, the qRT-PCR expression patterns of 19 out of the 20 randomly selected differentially expressed genes were in agreement with the results of the comparative transcriptome analysis with only slight differences in expression levels, indicating that there was no consistent bias in the expression patterns (i.e., in the direction of the differential expression or in the degree of fold change) for either method. Melting-curve analysis showed that a single product was amplified for all tested genes, indicating that the reference assembly was largely accurate and that it did not contain a large number of chimeric transcripts.

After carefully investigating the differentially expressed gene list from the skin tissues of XGC and YRC, we identified many putative pigmentation genes that may be associated with melanin synthesis, such as those encoding tyrosinase-related protein 1 (*TYRP1*), premelanosome protein (*SILV/PMEL*), cysteine/glutamate transporter (*xCT/SLC7a11*) and agouti signaling protein (*ASIP*). Melanin is the major pigment present in vertebrates [13]. There are two types of melanin, eumelanin and pheomelanin. Eumelanin corresponds to a brown/black color, while pheomelanin corresponds to a red/yellow color. As their names suggest, the skin color of XGC is red, while YRC is brownish-black, indicating that the amount and density of eumelanin are much lower in XGC skin than in YRC skin. Conversely, we hypothesized that the amount and density of pheomelanin might be much higher in XGC skin than in YRC skin. The observed differential expression patterns of melanin synthesis pathway genes endorsed this hypothesis. As shown in Table 3, the expression level of *TYRP1* was 5.7-fold up-regulated in YRC skin compared with XGC, suggesting that *TYRP1* is one key gene that contributes to brownish-black coloring in common carp by acting in the eumelanin synthesis pathway. *TYRP1* is expressed specifically in melanocytes, and it plays a crucial role in pigmentation and color patterning by affecting melanin synthesis, stabilizing tyrosinase protein, modulating tyrosinase catalytic activity, maintaining melanosome structure, and affecting melanocyte proliferation and cell death [14]–[16]. *TYRP1* was the first cloned pigmentation gene, and it was later mapped to the *brown* locus in mouse [17]. Mutations in *TYRP1* result in failure to form a black coat color, as observed in mouse [18], dog [19], cat [20], cow [21], and sheep [22]. In human cells, *TYRP1* is only detectable in cells containing eumelanin [23], and it is involved in the pigmentation differences among human populations [24]. These studies suggest essential roles for the *TYRP1* gene in eumelanogenesis. Similar roles for *TYRP1* have been reported in teleost fish. Knockdown of the *TYRP1* gene in zebrafish and medaka revealed that black eumelanin formation essentially relies on the presence of *TYRP1* [25]. Our results confirmed a role for *TYRP1* in the skin coloration of the common carp.

**Table 3:** Detailed information about the differentially expressed genes involved in each of the three pigmentation-related pathways.

Pathway involved	Gene name	Gene ID	Contig ID	Fold change
*Melanin biosynthesis*	*SILV*	ENSDARG00000091298	comp959125_c0_seq1	−5.2
	*ASIP*	ENSDARG00000077858	comp927400_c0_seq1	3.2
	*TYRP1*	ENSDARG00000056151	comp579560_c0_seq1	−5.7
	*xCT*	XP_697137.2	comp301533_c0_seq2	3.5
	*GNAQ*	ENSDARG00000011487	comp160287_c0_seq1	−6.7
	*ADCY2*	ENSDARG00000058392	comp168651_c0_seq3	6.6
*Wnt signaling*	*WNT5B*	ENSDARG00000034894	comp182251_c0_seq1	−2.5
	*SFRP5*	ENSDARG00000039041	comp236943_c0_seq1	−4.0
	*CAMK2*	ENSDARG00000021065	comp13326_c0_seq17	9.0
	*CTBP*	ENSDARG00000057007	comp10988_c0_seq11	−9.3
	*DAAM*	ENSDARG00000009689	comp55031_c0_seq10	3.3
	*EP300*	ENSDARG00000061108	comp7037_c0_seq5	7.8
	*FOSL1*	ENSDARG00000015355	comp243836_c0_seq1	2.7
	*MAP3K7*	ENSDARG00000020469	comp23078_c1_seq1	5.7
	*NKD*	ENSDARG00000055271	comp515909_c0_seq1	−2.9
	*NLK*	ENSDARG00000028793	comp19802_c0_seq2	7.9
	*PPP3C*	ENSDARG00000004988	comp41123_c0_seq1	−6.1
	*SENP2*	ENSDARG00000073837	comp25369_c0_seq2	−2.7
	*CCND1*	ENSDARG00000035750	comp126017_c0_seq1	−8.6
	*VANGL*	ENSDARG00000027397	comp129510_c0_seq1	−7.9
*MAPK signaling*	*FGFR2*	ENSDARG00000058115	comp87489_c0_seq3	−3.1
	*RAF1*	ENSDARG00000059406	comp47712_c0_seq2	8.1
	*TGFB1*	ENSDARG00000041502	comp132851_c0_seq7	−5.7
	*CDC42*	ENSDARG00000019383	comp80988_c0_seq8	−9.7
	*DUSP*	ENSDARG00000052465	comp125855_c0_seq11	−3.1
	*BRAF*	ENSDARG00000017661	comp118740_c0_seq2	−6.1
	*FOS*	ENSDARG00000031683	comp92067_c0_seq1	−2.2
	*MAP2K7*	ENSDARG00000008279	comp133947_c0_seq5	−7.7
	*MAPK8IP3*	ENSDARG00000062531	comp52280_c0_seq8	7.5
	*MAPKAPK3*	ENSDARG00000089016	comp9684_c0_seq1	2.5
	*PPP3C*	ENSDARG00000004988	comp41123_c0_seq1	−6.1
	*CACNA1C*	ENSDARG00000008398	comp125531_c0_seq1	−7.0
	*RASGRP3*	ENSDARG00000077864	comp144420_c0_seq5	−6.9
	*RPS6KA*	ENSDARG00000057927	comp9893_c0_seq6	8.4
	*SRF*	ENSDARG00000053918	comp11872_c0_seq1	−8.3
	*TAO*	ENSDARG00000079261	comp58691_c0_seq6	7.1
	*CACNB1*	ENSDARG00000002167	comp182816_c0_seq6	−6.3

Another significant eumelanin synthesis pathway gene we identified was *SILV/PMEL. SILV* encodes premelanosome protein, which is extensively expressed in pigment cells [26] and can catalyze the conversion of indole-5,6-quinone carboxylic acid into eumelanin [27]. Mutations in *SILV* causing pigmentation phenotypes have been reported in a number of vertebrate species. Inactivating *SILV* in mice led to a substantial reduction in eumelanin, indicating that *SILV* plays a critical role in maintaining efficient epidermal pigmentation [26]. In mice, *SILV* is required for normal melanosome development in skin melanocytes, choroid melanocytes and retinal pigment epithelium cells [26]. In the domesticated chicken, *SILV* mutations inhibited the production of all eumelanin in plumage and skin [28]. Similarly, in horses, *SILV* mutations caused a dilution of black pigment in the mane and tail [29]. Consistent with all these studies, our results showed that the expression level of *SILV* in YRC was significantly higher than in XGC, exhibiting a 5.2-fold up-regulation (Table 3). This result further confirmed the role of *SILV* in common carp eumelanogenesis.

Several other genes in our differentially expressed gene list are involved in the production of pheomelanin, the most significant of which are *ASIP* and *xCT*. In our results (Table 3), both *ASIP* and *xCT* were significantly up-regulated in XGC (by 3.2-fold and 3.5-fold, respectively), suggesting a significant effect on the production of pheomelanin in common carp. The functions of *ASIP* and *xCT* in pheomelanin synthesis have been widely studied in other species. ASIP can regulate pigmentation by antagonizing the binding of a-MSH to Mc1r, thus switching melanin synthesis from eumelanin to pheomelanin [30]. The ASIP protein also down-regulates genes such as *TYRP1*, *DCT*, and *TYR* to inhibit the synthesis of eumelanin [31], which is in agreement with our result that *ASIP* gene expression is up-regulated while *TYRP1* gene expression is down-regulated in XGC skin. The important role of *ASIP* in determining skin or coat color has been demonstrated in human [32], mouse [33], fox [34], rat [35], pig [36] and other species. *xCT* (*SLC7a11*), a cysteine/glutamate exchanger, mediates the cellular uptake of cysteine [37]. Cysteine is an important component of pheomelanin; therefore, *xCT* directly affects the pheomelanin synthesis pathway [38]. Loss of *xCT* expression caused marked inhibition of pheomelanogenesis [38]. Conversely, high-level expression of *xCT* contributed to the production of pheomelanin, as indicated by our results.

### Enrichment and pathway analysis

We attempted to categorize the 2,012 differentially expressed genes based on their likely functions by using gene annotation, GO term enrichment analysis, and KEGG pathway analysis. Using the Blast2GO software, all the differentially expressed genes were classified into different cellular, biological and functional gene ontologies. Together with the GO annotation of the reference sequences, the web-based program WEGO [39] revealed 17 significantly over-represented GO terms at the 2^nd^ GO level that were enriched in XGC skin relative to YRC skin. The top 10 of these are shown in Table 4, and they include pigmentation (GO:0043473), binding (GO:0005488), enzyme regulator activity (GO:0030234), biological regulation (GO:0065007), multicellular organism process (GO:0032501), multi-organism process (GO:0051704), developmental process (GO:0032502), cellular process (GO:0009987), localization (GO:0051179), and extracellular region part (GO:0044421). The primary interest of this study was to detect expression signatures indicative of fish skin pigmentation; therefore, the 363 genes in the pigmentation GO category were considered to be most informative for further pathway analysis.

**Table 4:** The top ten GO terms in the GO enrichment results of genes with significantly different expression in XGC skin compared to YRC skin.

GO ID	GO term	P value	Count
GO:0043473	Pigmentation	0.000	3706/363
GO:0005488	Binding	0.000	12707/1133
GO:0030234	Enzyme regulator activity	0.000	625/90
GO:0065007	Biological regulation	0.000	3912/390
GO:0032501	Multicellular organismal process	0.000	1609/173
GO:0051704	Multi-organism process	0.000	58/14
GO:0032502	Developmental process	0.002	1556/163
GO:0009987	Cellular process	0.004	8760/779
GO:0051179	Localization	0.008	2151/212
GO:0044421	Extracellular region part	0.012	301/38

The “Count” column indicates study count/population count; the study count is the number of genes associated with the GO term in the study set, and the population count is the number of genes associated with the GO term in the population set.

As described in a pathway analysis of non-model fish species [40], downstream pathway analysis was conducted using KEGG pathway analysis combined with manual literature searches. We mainly focused on pigmentation-related pathways to reveal the mechanism of skin color variation in the common carp. We included 1) melanin biogenesis; 2) the Wnt signaling pathway; and 3) the MAPK signaling pathway. Table 3 lists the key differentially expressed genes involved in each pathway.

**Figure 4:**
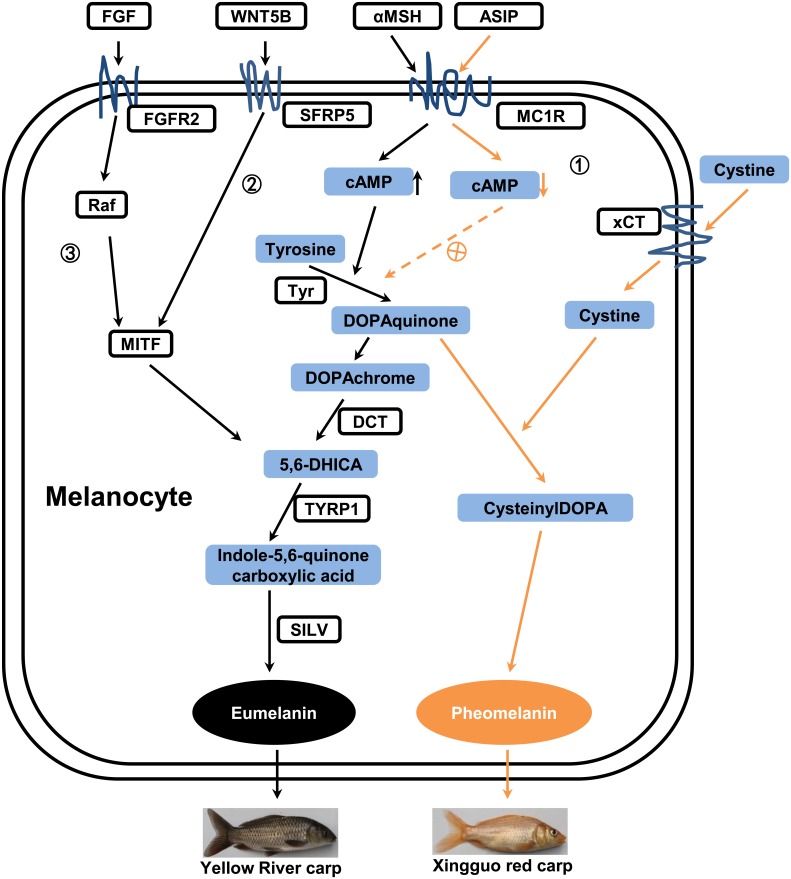
Diagram of putative gene pathways in the common carp skin pigmentation process. 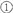
represents the melanin biosynthesis pathway. 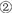
represents the Wnt signaling pathway. 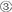
represents the MAPK signaling pathway.

Melanin biogenesis is under complex controls by multiple agents via pathways. Slominski et al. [41] elucidated that myriad hormonal factors are involved in regulating melanogenesis, such as melanocortins (MSH), ß-endorphin, endothelins, histamines, eicosanoids, catecholamines, c-kit ligand estrogens, androgens, vitamin D, serotonin, melatonin, dopamine, acetylcholine, agouti proteins and their receptors. Among those hormonal regulators, MSH with its receptor Mc1r is the most important positive regulators, whereas agouti proteins stand out among the negative regulators [41]. Besides hormonal regulators, nutritional regulators such as aromatic amino acid L-tyrosine and L-DOPA play critical roles in melanogenesis as well [42]. The molecular and genetic basis of pigmentation including the structure of tyrosinase, the development and differentiation of pigment cell, is largely conserved between mammals and teleost [41], [43]. The putative gene pathways involved in the common carp skin pigmentation process are shown in Figure 4, indicating the critical roles of the hormonal and nutritional regulators in melanogenetic pathways in common carp (Figure 4). Briefly, MSH binds to the G-protein-coupled receptor Mc1r, resulting in up-regulated cAMP levels, which in turn trigger the eumelanin biosynthesis process. The synthesis of eumelanin is then catalyzed successively by *Tyr*, *DCT*, *TYRP1* and *SILV* [44] (Figure 4). The switch from eumelanogenesis to pheomelanogenesis during melanin biosynthesis mainly depends on the presence of ASIP, an inverse agonist of Mc1r, which consequently make melanocytes start producing pheomelanin and stop producing eumelanin.

Signaling pathway such as Wnt and MAPK is implicated in numerous development and physiological process. Several studies have been reported that both Wnt and MAPK signaling pathways take part in the development of pigment cells [45]–[47]. Intriguingly, our results indicated that both pathways are highly likely to be involved in melanin biosynthesis in common carp. A group of identified differentially expressed genes involved in the Wnt or MAPK signaling pathway have the potential affecting the melanin biosynthesis (Table 3). For instance, *WNT5B*, a member of the Wnt protein family, is expressed in pigment cells located at the extended posterior edges of the caudal fin in zebrafish [48]. In our results, *WNT5B* was 2.5-fold up-regulated in YRC, suggesting that *WNT5B* may be involved in the skin pigmentation process. *SFRP5* (secreted frizzled-related protein 5), a member of the SFRP family that modulates Wnt signaling transduction, is highly expressed in the retinal pigment epithelium [49]. This finding, combined with our result that the expression level of *SFRP5* is higher in the skin of brownish-black YRC than in red XGC (Figure 3), raises the likelihood that *SFRP5* plays an important role in the Wnt signaling pathways involved in eumelanin synthesis.

The melanogenetic pathway in the skin could interact with other regulatory pathways such as nervous system, immune system, or circulatory system, via various organic compounds [50]. For instance, catecholamine, a compound produced in the skin, can be oxidized to form melanin, and also can affects the skin immune functions and systemic functions through communicating with specific cell surface receptors [50]. However, the details of interactions of regulatory pathways in common carp skin remain to be further investigated.

## Materials and Methods

### Ethics statement

This study was approved by the Animal Care and Use committee of the Centre for Applied Aquatic Genomics at the Chinese Academy of Fishery Sciences.

### Fish sampling, RNA isolation and sequencing

As described in our previous study [51], the two strains of common carp used in this study were sampled from distinct breeding populations. XGC individuals were collected from the National Fish Hatchery of Xingguo red carp, Xingguo, Jiangxi province, China. YRC individuals were collected from the Henan Academy of Fishery Sciences, Henan province, China. Six tissues from 18 individuals of each strain were collected; the tissues included brain, blood, gill, head kidney, skin and muscle. All tissues were placed in 2 ml of RNAlater (Qiagen, Hilden, Germany), and kept at −20°C until RNA extraction. RNA isolation was performed using TRIzol (Invitrogen, Carlsbad, CA, USA) with DNase I in accordance with the manufacturer’s instructions. RNA quality was verified using a Bioanalyzer 2100 (Agilent Technologies, Santa Clara, CA, USA). Equal amounts of high-quality RNA from each tissue were pooled and sent to HudsonAlpha Genomic Services Laboratory (Huntsville, AL, USA) for sequencing on an Illumina HiSeq2000 platform. All data generated were deposited into NIH Short Read Archive with accession number PRJNA254191.

### *De novo* assembly of reference sequences

All raw sequencing reads were trimmed by removing adaptor sequences, ambiguous nucleotides, low quality sequences (Q<20) and short reads (length below 30 bp) using CLC Genomics Workbench (CLC bio, Aarhus, Denmark). The Trinity software was then used to assemble all of the cleaned reads using default parameters. The assembled sequences were then filtered using the CD-hit program to reduce redundancy. The resulting contigs that were larger than 200 bp were considered as the final non-redundant transcripts and were used as the reference sequences.

### Transcriptome annotation and ontology

The assembled reference sequences were used as query sequences to search against the NCBI nr database, the UniProt database and the Ensembl zebrafish protein database. Searches were conducted using the BlastX program with an E-value cutoff of 1e^−10^. Gene ontology was performed by importing the zebrafish Blast results into Blast2GO software. GO terms were then assigned to each sequence automatically. The annotation output was categorized by cellular component, molecular function and biological process.

### Differential gene expression analysis

Clean reads from the skin tissues of each common carp strain were first aligned to their respective reference sequences using Bowtie 2 [52]. As described previously [53], RSEM was then used to calculate and estimate gene or isoform abundances. The expression level of each transcript in each sample was then normalized using edgeR [54]. Transcripts with fold change values larger than 2 and p values lower than 0.05 were included in subsequent analyses as the differentially expressed genes.

### qRT-PCR analysis

A total of 20 genes with significantly different expression in the two carp strains were selected for validation using qRT-PCR analysis. The house-keeping gene β-actin was used as an internal reference. All primer sequences are listed in Table S2. The first-strand cDNA was synthesized using the SuperScript III RT kit (Invitrogen) according to the manufacturer’s instructions. All cDNA samples were diluted to 100 ng/µl before use in qRT-PCR reactions on an ABI PRISM 7500 Real-Time Detection System (Life Technologies). The amplifications were performed in a total volume of 15 µl and included 7.5 µl of 2X SYBR Green Master Mix reagent, 1 µl of cDNA and 0.3 µl of each primer (10 µM). The thermal cycling profile consisted of an initial denaturation at 95°C for 10 min followed by 40 cycles of denaturation at 95°C for 15 s and annealing/extension at 60°C for 1 min. An additional temperature-ramping step from 95°C to 65°C was used to produce the melting curve. All reactions were conducted in triplicate and included negative controls with no template. Expression differences between XGC and YRC skin were assessed for statistical significance using a randomization test in REST software. The expression levels of genes were normalized to the levels of β-actin in the same sample.

### GO enrichment analysis

The web-based program WEGO was used with default parameters for statistical analysis of GO term overrepresentation among the genes with differential expression in the skin of the two carp strains. The study set represented the frequency of GO terms in the differentially expressed genes, while the population set corresponded to the whole reference gene set.

## Conclusions

To better understand the genetic mechanisms of skin color variation in common carp, we conducted a comparative analysis of the skin transcriptomes from two common carp strains with distinct skin colors, the red XGC and the brownish-black YRC. We detected 2,012 unique genes that were differentially expressed in the two strains. Further annotation, GO enrichment and pathway analysis indicated that key pigmentation-related genes were involved in at least three pathways that regulate pigment synthesis in the two strains. These results provide us with a valuable basis for understanding of the molecular mechanisms of skin pigmentation in teleost fish. Such understanding will facilitate the genetic selection and breeding of common carp with market-favored colors.

## Supporting Information

Table S1 **Detailed information of the differentially expressed genes.** The fold change cutoff is 2, and p-value cutoff is 0.05.
(XLSX)

Table S2 **Primers used for qRT-PCR validation.**
(XLSX)
